# Association between gene expression biomarkers of immunosuppression and blood transfusion in severely injured polytrauma patients

**DOI:** 10.1186/1757-7241-22-S1-O7

**Published:** 2014-07-07

**Authors:** Hew DT Torrance, Karim Brohi, Rupert M Pearse, Charles A Mein, Eva Wozniak, John R Prowle, Charles J Hinds, Michael J O’Dwyer

**Affiliations:** 1 Departments of Trauma and Critical Care, Royal London Hospital, Bart’s Health NHS Trust, London, E1 1BB, United Kingdom; 2 Bart’s & the London School of Medicine & Dentistry, Queen Mary University of London, London, E1 2AT, United Kingdom

## Background

Despite trauma patients using disproportionately large quantities of blood and blood products the immunomodulatory effects of blood transfusion in this group are poorly described.

## Methods

112 ventilated polytrauma patients were recruited in a London level 1 trauma centre. mRNA was extracted from PaxGene tubes collected within two hours of the trauma, at 24 hours and 72 hours. T helper cell subtype specific cytokines and transcription factors were quantified using real-time polymerase chain reaction.

## Results

Median Injury Severity Score (ISS) was 29. Blood transfusion was administered to 27 (24%) patients prior to the two hour sampling point. This was associated with a greater immediate rise in IL-10 (*p*=0.003) and IL-27 (*p*=0.04) mRNA levels.

Blood products were transfused in 72 (64%) patients within the first 24 hours. There was an association between transfusion within the first 24 hours and higher IL-10 (*p*<0.0001), lower Foxp3 (*p*=0.01), GATA3 (*p*=0.006) and RORyt (p=0.05) mRNA levels assayed at 24 hours. There were greater reductions in T-bet (*p*=0.03) mRNA levels and lesser increases in TNFα (*p*=0.015) and INFγ (p=0.035) assayed at 24 hours.

Multiple regression models confirmed that transfusion of blood products was independently associated with these early changes in gene expression.

Blood stream infections were seen in 15 (20.8%) of those patients receiving transfusions in the first 24 hours, compared to one patient (2.5%) in the group of those not transfused (OR 10.3 (1.3-81) *p*=0.008)).

## Conclusions

Polytrauma is associated with a primarily immunosuppressive inflammatory response, which was increased in magnitude in those patients receiving a transfusion of blood products. This may have important clinical consequences such as an increased susceptibility to develop serious nosocomial infections.

